# “I Am Ready and Willing to Provide the Service … Though My Religion Frowns on Abortion”—Ghanaian Midwives’ Mixed Attitudes to Abortion Services: A Qualitative Study

**DOI:** 10.3390/ijerph14121501

**Published:** 2017-12-04

**Authors:** Prince Oppong-Darko, Kwame Amponsa-Achiano, Elisabeth Darj

**Affiliations:** 1 Department of Public Health and Nursing, NTNU, Norwegian University of Science and Technology, 7491 Trondheim, Norway; elisabeth.darj@ntnu.no; 2 Public Health Division, Ghana Health Service, Accra, Ghana; kaash8@yahoo.com; 3 Department of Obstetrics and Gynecology, St Olav’s Hospital, 7030 Trondheim, Norway; 4 Department of Women’s and Children’s Health, Uppsala University, 75185 Uppsala, Sweden

**Keywords:** unsafe abortions, midwives perception, authorization, Ghana

## Abstract

Background: Unsafe abortion is a major preventable public health problem and contributes to high mortality among women. Ghana has ratified international conventions to prevent unwanted pregnancies and provide safe abortion services, legally authorizing midwives to provide induced abortion services in certain circumstances. Objective: The aim of the study was to understand midwives’ readiness to be involved in legal induced abortions, should the law become less restricted in Ghana. Methods: A qualitative study design, with a topic guide for individual in-depth interviews of selected midwives, was adopted. The interviews were tape-recorded and analyzed using content analysis. Results: Participants emphasized their willingness to reduce maternal mortalities, their experiences of maternal deaths, and their passion for the health of pregnant women. Knowledge of Ghana’s abortion law was generally low. Different views were expressed regarding readiness to engage in abortion services. Some expressed it as being sinful and against their religion to assist in abortion care, whilst others felt it was good to save the lives of women. Conclusion: The midwives made it clear that unsafe abortions are common, stigmatizing and contributing to maternal mortality, issues that must be addressed. They made various suggestions to reduce this preventable tragedy.

## 1. Introduction

Despite agreeing to adhere to international conventions, unsafe abortion remains a major public health challenge in Ghana [1]. The World Health Organization (WHO) defines unsafe abortion as a procedure for terminating an unintended pregnancy carried out either by persons lacking the necessary skills or in an environment that does not conform to minimal medical standards, or both. This definition comprises practices that create hazardous circumstances before, during, or after an abortion [2].

It is estimated that around 22 million unsafe abortions occur annually worldwide, and almost all of these are in developing countries [3]. Recent data estimate that 44,000 deaths occur due to unsafe abortion and that a disproportionate two-thirds of all abortion-related deaths occur in Africa [4].

Ghana has ratified numerous international charters and conventions, including the Universal Declaration of Human Rights and the United Nations Sustainable Development Goals (SDG). All countries are responsible for achieving SDGs, including the reduction of the global maternal mortality ratio to 70 per 100,000 live births by 2030, and unsafe abortion is one of the most common causes of maternal morbidity and mortality. However, many countries have laws that are generally against induced abortion, and abortion is only permitted to be performed under certain circumstances [3]. 

The Provincial National Defence Council Law, section 58 of Act 29 of 1960, amended 1985, regulates abortion in Ghana. The Act states that, “Abortion is unlawful and both the woman and anyone who abets the offence by facilitating the abortion by whatever means, are guilty of an offense of causing abortion” [1]. However, abortion is permitted under some circumstances: when pregnancy is the result of rape, defilement, or incest; if its continuation would involve risk to the life of the pregnant woman; if the pregnancy will injure the woman’s physical or mental health; or there is a substantial risk that the child may suffer from a serious physical abnormality or disease. Under any of these circumstances, abortion is permitted [1]. In 2006, the Ministry of Health and Ghana’s Health Service developed standards for the provision of comprehensive abortion care. Midwives have since then been authorized to provide early legal abortion in accordance with the law. The main idea behind the authorization was the shortage of physicians in the country. The WHO has established the minimum threshold of doctors, nurses, and midwives deemed necessary to deliver essential maternal and child health services at 23 per 10,000 people. Ghana, with limited numbers of health worker resources, currently has only 11 per 10,000, serving 28 million inhabitants [5]. The aim of shifting the task of performing abortions from physicians to midwives is to reduce the number of illegal and unsafe abortions. 

Realizing that authorized and well-trained midwives can provide competent and safe abortion-related services and that providing these safe services may reduce the amount of maternal deaths, governments have modified their laws and policies so as to include and empower midwives in providing abortion services [6] and some are considering the expansion of these services to include non-essential abortions. Unsafe abortions account for 11% of all maternal deaths, which is currently 319 per 100,000 live births in Ghana [5]. Midwives are trained as part of their curriculum to use manual vacuum aspiration (MVA) for post-abortion care in case of complications after miscarriage. However, little is known about midwives’ readiness and willingness to offer abortion services in Ghana. A successful expansion of the provision of safe abortion services would largely depend on the midwives and their motivation, knowledge, and readiness to provide abortion services. Studies have been performed in Ghana to explore the potential providers’ perceptions of abortion services [7], but none have specifically looked at midwives’ readiness to engage in these services, should the law be changed to make abortion less restricted. In order to frame the interpretation of the results from this present study, the socio-ecological model, first introduced by Bronfenbrenner, will be used in the discussion [8,9]. This model will make it easier to understand midwives’ views at different levels in society, as well as their own and others’ roles in preventing unnecessary and preventable maternal mortalities caused by unsafe and illegal abortions. The aim of this study was to explore midwives’ readiness for the provision of safe abortion services in Ghana.

## 2. Material and Methods

### 2.1. Study Design

A qualitative study design was used for the study. This design was chosen due to its ability to explore the participants’ attitudes and perceptions, through a prepared topic guide, covering areas of interest. Further, for a deeper understanding of issues, which were brought up by the respondents, their narratives were structured in subcategories and categories. A qualitative study aims to explore a phenomenon by answering questions of “how” instead of by counting numbers and answering questions of “what” or “how many” [10]. The midwives perceptions, experiences, and views would not have been able to catch using a quantitative survey. Individual in-depth interviews were preferred and employed, due to the sensitive nature of the topic. We anticipated that discussing potential illegal matters in a group could be a hindrance to obtaining open and in-depth information about their perceptions.

### 2.2. Study Setting

The study was conducted in a district in Western Ghana, with the majority of the inhabitants living in rural areas. The district has one government health center and nine smaller clinics.

### 2.3. Study Participants

The single inclusion criterion was that participants were practicing midwives in the district. All midwives were invited to take part voluntarily, with the aim of gaining a broad variation of views. No specific exclusion criteria were set in relation to age, gender, or length of work experience; however, no retired midwives were invited to participate. Mobile phone numbers for the eligible midwives were provided by the District Health Directorate. Text messages were sent to each midwife by the first author to inform them about the study. Each midwife was then called and asked whether they would like to receive more information about the study. If they agreed, they were sent printed copies of detailed information about the study, and a consent form. All written material was available in both English and Twi, the local language. Seven out of nine invited midwives agreed to take part in the study.

### 2.4. Data Collection

A convenient date and time for the interview was agreed with each of the midwives. Data for this study were collected using a semi-structured topic guide for individual in-depth interviews, covering areas such as the midwives’ views on their own profession regarding engagement in abortion services, their perceptions on the existing law, and their knowledge about the practices used for performing abortions. The interviews were conducted in Twi, a language that the participants were comfortable with and which was also spoken by the interviewer. The interviews were recorded using a voice recorder after seeking permission from the participants. The recorded audios were transcribed verbatim and translated into English for analysis, in order to include the non-Twi speaking collaborator.

### 2.5. Data Analysis

Content analysis, as described by Graneheim and Lundman, was used to analyze the data, in order to obtain the manifest meaning of the discussions [10]. Transcripts were read several times by the researchers, to allow them to become familiar with the text. Using Nvivo 11 (QRS International, Melbourne, Australia), the meaning units were condensed and coded. The coded groups were created into sub-categories, which were merged into categories, depending upon their associated findings. The categories and subcategories were discussed between the researchers until consensus was reached.

### 2.6. Ethical Reflection

This study was conducted in accordance with the Declaration of Helsinki, and the protocol was approved by the Regional Committee for Medical and Health Research Ethics (REK 2016/874) at NTNU, Norway, and the Ghana Health Service Ethical Review Committee. Consent was sanctioned from the District Directors of Health Services. All midwives gave their informed consent before they participated in the study. Participants were informed that, when the transcriptions of the recordings were made, the voice recording would be deleted, that it would not be possible to trace who said what during the individual interviews, and that results would only be provided at a group level. It was also made clear that, if they wanted to withdraw their consent, they could contact the researchers afterwards but before the analysis was completed. No monetary reimbursement was given to the participants.

## 3. Results

Seven practicing midwives participated, with ages ranging from 31 to 59 years. Their working experience ranged from 2 to 20 years. The individual midwives are referred to in the quotes as MW, with numbers 1–7. During the analysis, three main categories were identified, out of eight subcategories, from the individual in-depth interviews (Table 1).

### 3.1. Motivation to Be a Midwife

#### 3.1.1. Passion for Maternal Health

Midwives described how a strong desire for improving the health of pregnant women had motivated them to choose midwifery as a profession. They used the word “passion” and expressed the need that someone who is educated should care for women to avoid preventable ill health. They described experiences that had motivated them in their choice of profession. Living in a rural area had made them see the importance of having access to a health facility, especially to antenatal services. Furthermore, they were aware of positive outcomes where pregnant women were well cared for.

My main motivation is to help improve the health of pregnant women and to help save their lives.

*(MW2)*

I wanted to provide care for pregnant women. I have a passion for the health of pregnant women.

*(MW3)*

The midwives were highly aware of the high level of mortality that occurs among young pregnant women in Ghana and wished to improve the situation. The knowledge of these mortalities was a strong motivating factor for becoming a midwife and an issue that they wanted to help to reduce. They appreciated that their profession as educated midwives provided them with the possibility to assist in preventing maternal mortalities in dangerous situations.

I want to help reduce maternal mortalities.

*(MW4)*

I studied nursing before midwifery, so during my training as a nurse, I made up my mind that I needed to study midwifery so that I could help to reduce maternal mortalities.

*(MW1)*

Midwives expressed worries that maternal mortality is high in deprived areas of the country due to the apparent low access to antenatal services. They emphasized that pregnant women in rural communities without health facilities are at a high risk of maternal death, and they showed motivation and readiness to work in rural areas to help improve access to safe motherhood.

Women in rural areas who are usually poor do not have access to quality maternal health services during pregnancy. I did midwifery so that I could add to the few numbers of midwives so that maternal health will be accessible to pregnant women irrespective of where they live.

*(MW5)*

#### 3.1.2. Previous Experiences

These midwives were also community members and had been raised in rural areas. They had seen and heard of the dangers during pregnancies and deliveries and of the disastrous consequences after unsafe abortions. They were influenced by what they had learned and witnessed in the areas in which they lived during their childhood and youth. Mothers had told their daughters about maternal deaths, which they remembered. The study revealed that participants had become midwives due to such previous experiences. These participants revealed that they had heard several stories of maternal deaths in their community due to a lack of access to health care. This motivated them to become midwives so that they would help to save the lives of women in their communities.

When I was growing up I used to hear about women going into labor and dying, especially in the community I was living. My mother also told me about these issues. I also saw some myself that a woman … died. All [these] stories and what I heard encouraged me to study nursing and for that matter went ahead to study midwifery.

*(MW1)*

#### 3.1.3. Lack of Professional Opportunities

An alternative perception that was disclosed was that nothing special had motivated the participants to become midwives. Becoming a midwife was the only available opportunity and not necessarily the desired profession. They described how they wanted to get a professional job and secure their own income. They may initially have wanted to do something else and admitted that they became midwives only because they did not have the opportunity to pursue their desired career. However, as they expressed, they did not see any other possibility, and, given that they had been accepted on the midwifery education program, they had accepted this opportunity. Nothing came forward during the interviews about whether they now appreciated this choice or not.

For me, nothing motivated me, I was interested in becoming a disease control officer. I opted for midwifery when I did not get the opportunity to study disease control. I needed to develop my career and midwifery was the only available opportunity.

*(MW7)*

### 3.2. Unsafe Abortions Are Common and Hidden

#### 3.2.1. Stigmatization

Midwives admitted that unsafe abortion practices are common in the communities, and that living and working there, and having to take care of women with related complications made them well aware of it. However, abortions are hidden procedures, as it is perceived to be a taboo and not spoken about openly, except with very close friends, and hardly ever with health care providers. The midwives explained that society frowns on abortions and stigmatizes those who seek abortion services and those who provide the service alike.

It is something that goes on in the communities and because of the stigma attached to abortion, women who do abortions always do it under the cover of darkness.

*(MW7)*

… relating to unsafe abortions, it is unknown because of the way society perceives abortion. People hide to do it.

*(MW3)*

Another opinion expressed was that the extent of unsafe abortion was unknown, because victims only seek their help when there are complications. They said that they were not aware of the magnitude of illegal and unsafe abortions in society; however, they were regularly contacted when complications arose.

People do in the blindside of society, so it is something that goes on, but because of how society perceives it, it only comes to us when the victim suffers complications. When unsafe abortion is done and the victim does not suffer any complication, we do not hear about it.

*(MW5)*

#### 3.2.2. Unsafe Abortion Practices

The participants expressed worries about how pregnancies are terminated outside health facility settings. They emphasized that the methods used were dangerous and were the cause of complications of unsafe abortions. The midwives were aware of many crude ways in which women attempted to terminate pregnancies, such as the use of herbal mixtures to drink or as an enema, administering un-prescribed drugs, ground-up bottles mixed with Guinness beer, or the insertion of cassava sticks or herbs in the vagina. They described how the use of misoprostol (Cytotec^®^), normally used in postpartum hemorrhages, was known and used illegally for the termination of a pregnancy.

Some prepare herbal concoction and get it into the body through enema. The herbal concoction then forces the fetus to fall out. Some also use grinded bottles mixed with Guinness for enema. This is very dangerous!

*(MW5)*

Some use enema or drink all kinds of herbal concoctions, some insert Cytotec into the vagina; yes, they know about Cytotec! Some also drink un-prescribed drugs which they buy from the local drug stores.

*(MW7)*

Some insert herbs in the vagina. Some also use sticks; cassava stick, they insert it into the vagina.

*(MW6)*

### 3.3. The Law and Abortion

#### 3.3.1. Knowledge and Ignorance of Existing Law

As described above, abortion is a crime under the laws of Ghana, and only permitted under certain circumstances. A common misunderstanding revealed in the interviews was concerning the legality of performing an induced abortion. Participating midwives could not demonstrate a good understanding of the abortion laws and some misinterpreted the nature of Ghana’s abortion laws, while others described that they understood all abortions to be legal in Ghana. This interpretation was apparent in both old and young midwives.

I know that abortion is legal under the law, so if someone walks in and you are trained, you can do it for the woman. Also, if a pregnant woman attempts an unsafe abortion, you can complete it for her.

*(MW5)*

Abortion is legal in Ghana. If you get pregnant and you feel you do not want it, the law allows you to terminate the pregnancy.

*(MW1)*

#### 3.3.2. Views on the Current Abortion Law

There was a general perception that legalizing abortion and making it accessible would increase the number of abortions that occur in the country. The participants who had the correct knowledge and perceptions of Ghana’s abortion law thought that the law was satisfactory in its current state. They described how they thought that abortions should not be wholly legalized.

I do not support the termination of any pregnancy outside what the law allows. I feel that the law is good.

*(MW7)*

Providing safe abortion services should be done within the remit of the law. It should not like abortions to be allowed for every unwanted pregnancy. But if it affects the health of the pregnant women, then why not! Such a pregnancy can be terminated and that is what the law allows.

*(MW4)*

#### 3.3.3. Willingness to Provide Safe Abortion Care

The participants expressed mixed views about whether safe abortion care should be offered, assuming it was legalized and could be provided on maternal request. Despite their concerns about maternal mortalities and the habit of performing unsafe, hidden abortions, expressions came forward that they felt abortion was against their religious beliefs—that it was sinful and they did not want to be involved. Those who shared this view were, however, ready to help in situations of incomplete abortions and miscarriages.

I am not ready and I am not willing to provide comprehensive abortion services where a woman can just walk to me and ask for an unwanted pregnancy to be terminated. The best I may offer is when the woman comes with an incomplete abortion, in that case, I will help complete the abortion for her … I would rather refer a woman who needs comprehensive abortion care to the nearest health facility where the service is provided.

*(MW1)*

Seriously for me, it is against my religious belief and that does not allow me to provide abortion services. I can provide clients with pre-abortion counselling, but not to do the actual abortion.

*(MW4)*

Others felt that, although abortion was against their religious beliefs, they were ready to provide abortion care within the remit of the law, in order to save lives. They were of the opinion that it is always better to save lives than to allow the women to perish from preventable causes.

Providing safe abortion services and making it readily available and accessible is one way of preventing maternal deaths. For me, I am ready and willing to provide the service … though my religion frowns on abortion, but I see this profession as a duty call, devoid of religious and moral judgement. It’s more important that our women do not die from these kinds of avoidable deaths.

*(MW2)*

## 4. Discussion

It is an undeniable fact that unsafe abortions are one of the main causes of maternal mortality and morbidity [2,4]. In order to save women’s lives, there is an urgent need to address the setbacks that hinder the progress of improving maternal health in the developing world. In this study, we focus on midwives’ views of their potential role in the prevention of unsafe abortions and its consequences. Our results relating to the perceptions of a willingness to diminish inequality are seen from the perspective of the socio-ecologic theoretical framework, initially described by Bronfenbrenner and developed by others into four different levels: individual, relationship, community, and policy levels [11,12].

At an individual level, the midwives are concerned about the consequences of unsafe abortions. They have experiences of maternal deaths and were motivated to save lives, but simultaneously they have mixed feelings about personally engaging in abortion services. They are well aware of the illegal methods and procedures that are used, as they have a duty to deal with any complications that arise. Despite their awareness and concerns, they expressed a reluctance to participate in actual comprehensive abortion services; however, they did not object to providing post-abortion care when this was needed. They were more willing to help by referring women to the nearest health facility where abortion services were available and related this to their personal religious beliefs. This finding is also revealed in another study, where various health care professionals were interviewed and expressed conflicting dilemmas between their religious and moral beliefs about the sanctity of life and their duty to provide safe abortion care [7]. This attitude is likely to hinder midwives from providing safe abortion services. However, abortion is a sensitive topic worldwide; it raises ethical, cultural, moral, and religious questions wherever it is debated, and strong personal beliefs contribute to opposing the procedure, as revealed in research from South Africa [13]. Religious beliefs did not prevent some of the interviewed midwives from providing abortion care; however, as they perceived their profession as a duty and need to help prevent deaths from unsafe abortions, this overruled their personal feelings.

The American College of Nurse-Midwives conducted a survey among their members to determine their attitudes towards abortions, and the results showed that 79% supported unlimited access to abortion and that 52% supported abortion practice by nurse-midwives [14]. However, in another study, abortion procedures have been known to be hindered due to a lack of nurses who are willing to assist physicians with the procedure [15].

Regarding the relationship level, couples need to be able to plan the size of their families and to decide whether and when to have a child, as well as the age gap between children. Having access to modern contraceptives is important, as it will decrease the amount of unwanted pregnancies and, consequently, unsafe abortions [4]. This will further reduce the burden of the ethical dilemma that the midwives expressed. Smaller family sizes will reduce poverty, increase financial capital within the family, and may give children the possibility of participating in higher levels of education than their parents did. However, in many developing countries, fertile women are less likely to use modern contraceptives [16]. Three out of every 10 women of reproductive age in Ghana had an unmet need for contraception in 2014 [17]. A Greek study shows that cultural differences significantly affect contraceptive behavior. Nevertheless, interventions that promote contraception can still be successful in other populations [18]. Thus, contraceptives should be made readily accessible, affordable, and acceptable for use by people in relationships.

In relation to the community level, midwives in this study chose the profession because they wanted to care for pregnant women in their communities. They have much concern for reducing maternal deaths, a finding that is similar to those of a study in Papua New Guinea, in which midwifery students expressed the same motivation for choosing their profession [19]. As a result, they experience frustration about the number of unsafe abortions in their communities, which contribute to maternal losses. However, abortions do evoke religious, moral, ethical, and medical concerns. Abortion can be highly stigmatizing and poses challenges; both to the pregnant women, and health care providers and researchers. The midwives expressed that pregnant women were stigmatized in the community, whether they just talked about it or had an abortion [6]. Another study has reported similar negative attitudes among health providers towards women seeking abortions, and these are frequently driven by socio-cultural and religious norms, and contribute further to the stigmatization [7]. Midwives in this study felt that they were also frowned upon if they engaged in this service. However, where health care providers were exposed to higher levels of education, including training overseas, this seemed to result in more positive, less stigmatizing views towards the need for safe abortion services [7].

On a policy level, the health system has the responsibility to provide policies and legislation with the aim to improve and ensure the health and well-being of the people of Ghana. The legalization of the safe abortion care service is a human rights imperative, and making abortions accessible and affordable, together with improved access to contraceptives, will be an important step towards reducing the high maternal mortality ratio in Ghana. Studies report that health professionals who do not support safe abortion often lack sufficient knowledge of current legislation in their countries [20]. Health centers are the first level of primary care in rural areas, and these are usually managed by a midwife or a trained community health nurse. This study showed that midwives had limited understanding of the abortion law in Ghana, and could not clearly state the position of the law regarding safe abortion care. Contrary to this law, some midwives believe that abortion is fully legalized and hence, any person who does not want a pregnancy can request abortion at any health facility and be provided a safe service. This finding contradicts the high level of knowledge found among doctors in a similar study in Ghana [1]. Although some participants showed strong feelings about unsafe abortions and its consequences, any modification of the abortion law to make it legal was, for some, not appreciated. The successful implementation of any health sector reform is dependent on the acceptance and willingness of health professionals to implement the desired change, and midwives can play a key role in this. 

There was a fear among the midwives in this study that the numbers of unsafe abortions would increase if the laws were less restrictive; however, legalization in other countries has not confirmed this concern [21]. Haddad and Nour, in their review of unsafe abortion globally, demonstrated that liberalizing abortion laws is correlated with a reduction in the rate of abortion-related morbidity and mortality [21]. However, these studies are mainly from high-income countries, as similar studies are scarce from low-income countries. When abortions are performed by skilled providers using appropriate medical techniques and drugs, and under hygienic conditions, induced abortion is a safe medical procedure. A remarkable decline in the abortion rate has been seen in developed countries where abortion is available on request and contraception readily accessible [21,22,23].

However, less restrictive abortion laws do not guarantee safe abortions for all in need. In India, despite abortions being legal, women with low levels of education still turn to unqualified local providers for abortion, if access is limited [24]. Complications arising from unsafe abortions are common, which the midwives in this study were very much aware of. Estimates from 2012 indicate that 6.9 million women in developing regions were treated for complications from unsafe abortions [4]. The treatment of medical complications places a considerable financial burden on women and on the public health care systems, which have limited resources and funds to devote to health services [4].

### 4.1. Trustworthiness

Measures were taken to promote the trustworthiness of this study [25]. Credibility was ensured as the study was undertaken by two Ghanaian researchers, both of whom work within the public health sector, are fluent in the Twi language, and are familiar with the study settings. The international researcher, an obstetrician, has experience of conducting reproductive health research in Africa and other developing regions, and is knowledgeable in qualitative research methods. Using a semi-structured interview guide, audio recording the interviews, providing verbatim transcription, and employing a systematic process of data analysis enhanced the confirmability of the results. Transferability was achieved by providing a detailed description of the methodology and setting. In addition to the detailed description of the methods, the same researcher conducted all seven individual in-depth interviews within a period of one month to increase the consistency of data and to achieve dependability.

### 4.2. Strengths and Limitations of the Study

The strength of this study is the qualitative approach, as, to our knowledge, no such study has previously been performed among midwives in Ghana. In order to structure the findings we used the socio-ecological model, which was suitable as it argues that individual behavior is shaped by factors at multiple levels, including individual, community, and policy levels in addition to intrapersonal and interpersonal levels [9]. A strength of this study was that interviews were performed individually, as using FGDs was anticipated as having the potential to deter the participants from disclosing their personal views on this sensitive matter.

A limitation could be that only nine midwives were available in the chosen district. More information may have been achieved if more midwives were available, or if a second district was included. However, the researcher felt that the midwives spoke freely and openly about their feeling and perceptions regarding abortions, and no more information came up after the sixth interview. One more interview was made to ensure the saturation of the material. More information may have surfaced if doctors and community members had been included in this study, but this was not the aim of this first study.

## 5. Conclusions

By interviewing practicing midwives in primary health care, we found that a change in the current Ghanaian abortion law to make abortion legal and to make the service available and safe, would need clarification across the health system and the provision of training and leadership support to the providers in the health facilities. As there is a shortage of physicians in the country, midwives will be the potential future providers of comprehensive abortion care, in order to reduce the tragic and preventable deaths among pregnant women who choose a dangerous, unsafe method to terminate an unwanted pregnancy. Midwives will need support, guidelines, supervision, orientation on the behavior necessary to boost confidence in providing safe abortion care, further repeated training, and the necessary equipment.

The community should be made aware of the knowledge that health providers already have: that the high numbers of maternal deaths in Ghana are partly because of unsafe abortions. Awareness of the possibility of saving lives if abortions were legal and discussion with stakeholders are necessary steps towards making a change. Further, the Ghanaian government needs to promote and make known the declarations that have already been signed and the responsibility that has been guaranteed in order to follow the international standards and the United Nations Sustainable Development Goals.

Guidelines must be disseminated and support given to the primary health providers for giving Ghanaian women access to safe health care, including abortions. Simultaneously, it is important that policy-makers not only make abortion legal but also make family planning methods accessible, as this has been proven to be one of the successes that contributes to reducing the amount of abortions in other countries.

## Figures and Tables

**Table 1:** Categories and subcategories from participants’ responses.

Categories	Subcategories
Motivation to be a midwife	Passion for maternal health
	Previous experiences
	Lack of professional opportunities
Unsafe abortions common and hidden	Stigmatization
	Unsafe abortion practices
The law and abortion	Knowledge and ignorance of existing law
	Views on abortion law
	Willingness to provide safe abortion care
